# Silencing Long Non-coding RNA Kcnq1ot1 Limits Acute Kidney Injury by Promoting miR-204-5p and Blocking the Activation of NLRP3 Inflammasome

**DOI:** 10.3389/fphys.2021.721524

**Published:** 2021-11-11

**Authors:** JunTao Wang, Peng Jiao, XiaoYing Wei, Yun Zhou

**Affiliations:** ^1^Department of Nephrology, The First People’s Hospital of Shangqiu, Shangqiu, China; ^2^Department of Emergency, The First People’s Hospital of Shangqiu, Shangqiu, China; ^3^Institute of Nephrology Eastern Theater General Hospital, Nanjing, China

**Keywords:** lncRNA Kcnq1ot1, competitive endogenous RNA, miR-204-5p, NLRP3, inflammasome, lipopolysaccharide, acute kidney injury

## Abstract

Acute kidney injury (AKI) is a critical clinical disease characterized by an acute decrease in renal function. Long non-coding RNAs (LncRNAs) are important in AKI. This study aimed to explore the mechanism of lncRNA Kcnq1ot1 in AKI by sponging microRNA (miR)-204-5p as a competitive endogenous RNA (ceRNA). AKI mouse model and hypoxia/reoxygenation (H/R) model of human kidney (HK) cells were established. Kcnq1ot1 expression, cell proliferation, and apoptosis were measured. Binding relations among Kcnq1ot1, miR-204-5p, and NLRP3 were verified. Pathological changes and cell apoptosis were detected. The results showed that Kcnq1ot1 was highly expressed in the AKI model *in vivo* and *in vitro*. Kcnq1ot1 knockdown promoted cell proliferation and prevented apoptosis and inflammation. Furthermore, Kcnq1ot1 inhibited miR-204-5p expression by competitively binding to miR-204-5p in HK-2 cells. miR-204-5p targeted NLRP3 and NLRP3 overexpression averted the inhibiting effect of miR-204-5p on apoptosis and inflammation in HK-2 cells *in vitro*. Kcnq1ot1 knockdown *in vivo* promoted miR-204-5p expression, inhibited NLRP3 inflammasome activation, reduced levels of SCr, BUN, and KIM-1, and thus alleviated AKI and reduced apoptosis. In summary, silencing lncRNA Kcnq1ot1 inhibited AKI by promoting miR-204-5p and inhibiting NLRP3 inflammasome activation.

## Introduction

Acute kidney injury (AKI), also known as acute renal failure, is a group of clinical conditions characterized by sudden decreases in serum creatinine (SCr) and urine output which suggest a rapid drop in renal function-glomerular filtration rate (Levey and James, 2017; Ronco et al., 2019). The incidence of AKI has been increasing recently and the morbidity and mortality rates remain high (Negi et al., 2018). The etiologies of AKI can be classified into pre-renal types, such as diminished renal perfusion and pre-renal azotemia, intra-renal type, and post-renal type, such as acute post-renal obstructive nephropathy (Koza, 2016; Gu et al., 2020; Roy and Devarajan, 2020). AKI could lead to long-term outcomes including chronic kidney diseases or end-stage kidney diseases and cardiovascular conditions (Kashani et al., 2017). Currently, no interventions are available, and thus successful and sustainable care bundles are needed for AKI prevention and management (Vanmassenhove et al., 2017; Moore et al., 2018). As a result, the exploration of therapeutic targets for AKI is important and in urgent need.

Long non-coding RNAs (lncRNAs), which might serve as the potential molecular targets in treating AKI, are long RNA transcripts longer than 200 nucleotides that are not translated into proteins (Dahariya et al., 2019). LncRNAs exhibit tissue-specific expression patterns and play an essential role in cellular biology and various diseases (Ignarski et al., 2019), including AKI (Zhou et al., 2019). For example, overexpression of lncRNA HOX transcript antisense RNA alleviates AKI in sepsis rats by inhibiting the apoptosis of kidney cells by regulating the miR-34a/Bcl-2 pathway (Jiang et al., 2019). LINC00963 promotes AKI progression by targeting miR-128-3p to reduce apoptosis (Xie et al., 2020). Silencing lncRNA Kcnq1ot1 attenuates neuroinflammation and neuron apoptosis induced by lipopolysaccharide (LPS) *via* sponging miR-30e-3p (Song et al., 2021). Silencing Kcnq1ot1 suppresses expressions of inflammatory cytokines and apoptosis (Zhao et al., 2021). The intervention of lncRNA Kcnq1ot1 reduces the endoplasmic reticulum stress and inhibits cell apoptosis in cerebral ischemia/reperfusion injury by targeting miR-30b/GRP78 (Li Y. et al., 2020). But the role of lncRNA Kcnq1ot1 in AKI has not been demonstrated in former studies.

Competitive endogenous RNAs (ceRNAs) comprise protein-coding RNA, tRNA, rRNA, lncRNA, pseudogene RNA, and circular RNA (An et al., 2017). Long non-coding RNAs Kcnq1ot1 accelerates atherosclerosis via acting as a ceRNA (Yu et al., 2020). Moreover, microRNAs (miRNAs) are differentially expressed in AKI and play pivotal roles in the development of AKI (Zou and Zhang, 2018). Kcnq1ot1 was reported to bind to miR-204-5p to manipulate myocardial injury in mice (Rong et al., 2020). miR-204-5p overexpression alleviates renal ischemia/reperfusion (I/R) injury in mice by blocking the Fas/FasL pathway (Zhu et al., 2019). The NLR pyrin domain-containing 3 (NLRP3) inflammasome is implicated in the progression of AKI and other kidney diseases (Hutton et al., 2016). Therefore, we speculated that Kcnq1ot1 may regulate inflammation in AKI by sponging miR-204-5p as a ceRNA to modulate the NLRP3 inflammasome.

## Materials and Methods

### Ethics Statement

This experiment obtained the approval from the Animal Care and Use Committee of The First People’s Hospital of Shangqiu (Approval number: 2020-008), and animal experiments followed the Guide for the Care and Use of Laboratory Animals.

### Establishment of Acute Kidney Injury Mouse Model

A total of 50 C57BL/6J mice (aged 10-12 weeks; Hunan SJA Laboratory Animal Co., Ltd., Changsha, China) were included. There were 10 mice randomly assigned to a normal control group (without any treatment). Then, 10 mice were randomly assigned to a sham group, and the rest of the 30 mice were used to establish the AKI model and randomly assigned to 3 groups (*N* = 10), which are ischemia/reperfusion (I/R) group (30 min of bilateral ischemia), sh-NC group (injected slowly with a total volume of 20 μl sh-NC adenovirus with a titer of 1 × 10^7^ particles/μl *via* the caudal vein using a 4-gauge needle 1 week before I/R treatment), and sh-Kcnq1ot1 group (injected with 20 μl sh-Kcnq1ot1 adenovirus with 1 × 10^7^ particles/μl via the caudal vein 1 week before I/R treatment). The sh-Kcnq1ot1 or sh-NC (sh-Kcnq1ot1 sequence: 5′-GCAGAACCAUCGAUGGUGCGU-3′; sh-NC: 5′-AGUGCUGCGCACGUGUCUCAU-3′; RiboBio, Beijing, China) was introduced into the adenoviral vector (Life Technologies, Shanghai, China) to form adenovirus solution of sh-Kcnq1ot1 or sh-NC by using Gateway^TM^ LR Clonase II Enzyme Mix (Invitrogen, Carlsbad, CA, United States) (Xue et al., 2021). Mice were intraperitoneally injected with pentobarbital sodium (60 mg/kg; Sigma-Aldrich, St. Louis, MO, United States) to ensure that mice were fully anesthetized before the surgery and a heating pad was used to maintain the body temperature of mice.

Laparotomy was conducted to check the renal pedicle. The renal pedicle was occluded with a microvascular clamp for 30 min to induce bilateral renal ischemia. Sham procedures were performed similarly without clamping the bilateral renal pedicles. The mice were hydrated with warm saline and the body temperature was maintained constantly at 37°C using a heating pad until awake. The wounds were sutured after removing the clamps and the mice were allowed to recover. All mice were euthanized after 2 h of reperfusion. After taking a.5 ml blood sample from the orbit, the kidney tissues were collected, followed by further analysis of the blood sample and kidney tissues (Lempiainen et al., 2012; Xue et al., 2021). The kidney tissues of five mice in each group were fixed with 4% paraformaldehyde for 8 h, dehydrated with conventional alcohol, cleared with xylene, embedded in paraffin, and cut into 5 μm for hematoxylin and eosin (HE) staining and terminal deoxynucleotidyl transferase dUTP nick end labeling (TUNEL) staining. The kidney tissues of five mice in each group were stored in the cryopreservation tubes, put into liquid nitrogen (LN) for rapid freezing, and transferred to an ultra-low temperature refrigerator for molecular biological detection.

### Enzyme-Linked Immunosorbent Assay

The levels of SCr and blood urea nitrogen (BUN) were tested using a Beckman Autoanalyzer (Beckman Coulter, Fullerton, CA, United States). An ELISA Kit from Cosmo Bio (Tokyo, Japan) and an ELISA Kit from Sangon Biotech (Shanghai, China) were, respectively, used to examine the urine concentration of kidney injury molecule 1 (KIM-1) and the cultured concentration of tumor necrosis factor-α (TNF-α), interleukin (IL)-6, IL-10, and IL-1β (Yuan et al., 2021).

### Hematoxylin and Eosin Staining

After rinsing with normal saline, the kidney tissue samples were fixed for 30–50 min in 4% paraformaldehyde, followed by rinsing, dehydration, clearing, waxing, embedding, and sectioning. The tissues were flattened and placed on the slide and dried in a constant temperature box for 45°C. Then, the tissues were dewaxed, rehydrated through high concentration to low concentration ethanol, and then rinsed with distilled water for 5 min. Subsequently, the tissue sections were stained for 5 min with hematoxylin solution, washed with running water for 3 s, differentiated with 1% ethanol hydrochloric acid (HCI) for 3 s, and stained with 5% eosin solution for 3 min, followed by dehydration, clearing, and sealing. A microscope (DM750, Leica; Beijing Jiayuan Industrial Technology Co., Ltd, Beijing, China) was used to observe tissue sections. Acute tubular necrosis (ATN) scoring system was adopted to analyze pathological injury. The tissue damage was quantified in a blinded manner and scored according to the percentage of damaged tubules in the sample: 0, no damage; 1, <25% damage; 2, 25–50% damage; 3, 50–75% damage; 4, >75% damage (Lempiainen et al., 2012; Xue et al., 2021).

### Transferase dUTP Nick End Labeling Staining

Frozen sections of kidney tissues were incubated with phosphate buffer saline (PBS) containing 0.1% Triton X-100 (PS0016, Leagene Biotechnology, Beijing, China) for 5 min. The following experimental procedures were conducted according to the TUNEL assay kit (C1090, Beyotime, Shanghai, China). The TUNEL assay solution was prepared (TdT enzyme: fluorescent marker solution = 1:9) and thoroughly mixed. The cell surface was rinsed with PBS once, and each sample was added with 50 μl TUNEL assay solution and incubated at 37°C for 60 min without light exposure. Afterward, the staining solution was removed, and cells were rinsed with PBS three times. After sealing with anti-fluorescence quenching solution, the cells were observed with a fluorescence microscope (Leica SR GSD, Leica, Wetzlar, Germany). According to the distribution of apoptotic cells, five positive visual fields were selected from each section under a light microscope at 200× magnification, and 200 cells were counted in each visual field. The percentage of average apoptotic cells was the apoptotic index.

### Reverse Transcription-Quantitative Polymerase Chain Reaction

The total RNA was isolated from tissues and cells using a TRIzol kit (Invitrogen, Carlsbad, CA, United States). The concentration and purity of total RNA were measured using a NanoDrop2000 ultraviolet microspectrophotometer (NanodDop Technologies, Wilmington, DE, United States). Reverse transcription was performed to generate cDNA using TaqMan MicroRNA Assays Reverse Transcription primer (4427975, ABI, Foster City, CA, United States). The primers of Kcnq1ot1, miR-204-5p, and NLRP3 were designed and synthesized by TaKaRa (Shiga, Japan) (Table 1). Real-time fluorescence qPCR was carried out using an ABI7500 quantitative PCR instrument (ABI). The reaction conditions were pre-denaturation at 95°C for 10 min and a total of 40 cycles of denaturation at 95°C for 10 s, annealing at 60°C for 20 s and extension at 72°C for 34 s. Glyceraldehyde 3-phosphate dehydrogenase (GAPDH) was used as a control for Kcnq1ot1 and NLRP3, and U6 was used as a control for miR-204-5p. The 2^–ΔΔCt^ method was employed to calculate the relative transcription level of target genes (Ayuk et al., 2016). Each experiment was repeated three times.

**TABLE 1:** Primer sequences.

**Primers**	**Sequences**
*Kcnq1ot1*	F:5′-CGCGATCCTCCTCAGTGTTT-3′
	R:5′-CATATCGCCGACCACCATGA-3′
*miR-204-5p*	F:5′-ACACTCCAGCTGGGTTCCCTTTGTCATCCTAT-3′
	R:5′-CTCAACTGGTGTCGTGGAGTCGGCAATTCAGTTG AGAGGCATAG-3′
*NLRP3*	F:5′-AGATGCTGGAATTAGACAACTG-3′
	R:5′-CATTTCACCCAACTGTAGGC-3′
GAPDH	F:5′-CCACCCAGAAGACTGTGGAT-3′
	R:5′-TTCAGCTCAGGGATGACCTT-3′
U6	F:5′-CTCGCTTCGGCAGCACA-3′
	R:5′-AACGCTTCACGAATTTGCGT-3′

*Kcnq1ot1, KCNQ1 overlapping transcript 1; miR, microRNA; NLRP3, Nod-like receptor protein 3; GAPDH, glyceraldehyde-3-phosphate dehydrogenase; F, forward; R, reverse.*

### Western Blot

The tissues placed into centrifuge tubes were added with 100 μl radioimmunoprecipitation assay (RIPA) lysis buffer (R0020, Solarbio, Beijing, China) which contained 1 mmol/L phenylmethylsulfonyl fluoride (PMSF; added as required), followed by homogenization at 3,000 r/min until being fully cracked. Then, the tissues were centrifuged at 4°C at 12,000 *g* for 4 min after standing 30 min on ice. The supernatants were removed and conserved at –80°C. Protein concentration was detected using the bicinchoninic acid kit (AR0146, Boster, Wuhan, Hubei, China) and adjusted to 3 μg/μl. The protein was added with loading buffer and boiled for 10 min at 95°C. Then, 30 μg protein samples were loaded in each well, followed by separation with polyacrylamide gel electrophoresis. The protein was then transferred onto the polyvinylidene fluoride (PVDF) membranes (P248, Sigma-Aldrich) using a semi-dry transfer method. After blocking for 1 h with 5% bovine serum albumin (10-L16, Zhongsheng Likang, Beijing, China), the membranes were incubated with rabbit anti-NLRP3 (ab263899, 1:1000), caspase1 (ab138483, 1:1000), and ASC (ab155970, 1:5000) at 4°C overnight. Then, the membranes were rinsed with Tris-buffered saline-Tween-20 (TBST) (T1081–500 ml, Solarbio) in triplicate (5 min per rinse) and added with goat anti-rabbit secondary antibody (ab6721, 1:10000, Abcam, Cambridge, United Kingdom) for 1 h incubation and rinsed with TBST in triplicate (5 min per rinse). The blots were developed using a chemiluminescence reagent (abs920, Absin Bioscience Inc., Shanghai, China). GAPDH (ab181602, 1:10000) was used as an internal control. Bio-rad Gel Dol EZ imager (Bio-Rad, Hercules, CA, United States) was utilized for development. ImageJ software 1.48 (National Institute of Health, Bethesda, MD, United States) was employed for gray value analysis of the target bands. The experiment was conducted in triplicate.

### Establishment of H/R Cell Models

Human kidney-2 (HK-2) and human embryonic kidney (HEK) 293 cell lines (ATCC, Manassas, VA, United States) were cultured in RPMI-1640 medium with 10% heat-inactivated fetal bovine serum (FBS) and 1% penicillin-streptomycin (Sigma-Aldrich) at 37°C with 5% CO_2_, and humidified atmosphere. For the H/R treatment, HK-2 cells were cultured in the medium with low-glucose concentration for 48 h and removed. After two washes with PBS, HK-2 cells were treated with inhalation of pure oxygen (O) (100% O) for 15 min and placed in an airtight container with 95% N_2_ and 5% CO_2_ for 3 h. Finally, HK-2 cells were reoxygenated (95% air and 5% CO_2_) after the addition of fresh low-glucose Dulbecco’s modified Eagle’s medium with 10% FBS at 37°C, for a total of 3 h of reoxygenation. The H/R model was used for the functional experiments (Yuan et al., 2021). The sh-Kcnq1ot1 and its control plasmids, miR-204-5p mimic and its control plasmid, and NLRP3 overexpression vector and its control plasmid were purchased from GenePharma (Shanghai, China) and transfected into the cells at the final concentration of 50 nM in accordance with the instructions of Lipofectamine 2000 (11668-019, Invitrogen). The culture medium was changed 6 h after transfection and cells were collected after 24–48 h for subsequent experiments. Each experiment was repeated 3 times independently.

### Cell Counting Kit-8 Assay

Cells were seeded into 96-well plates (1 × 10^3^ cells/well) in 100 μl medium which contained 10% FBS and cultured for 1–5 days. Cells were counted using the Cell Counting Kit-8 (CCK-8) kit (Dojindo, Japan). Next, 10 μl CCK-8 solution was added to each well and incubated for 1 h. Absorbance at 490 nm was detected using a microplate reader.

### Flow Cytometry

Cell apoptosis was measured using Annexin V-FITC/PI staining method. Cells were detached using.25% trypsin (without EDTA; YB15050057, Yubo Biotech, Shanghai, China), collected and centrifuged, and supernatants were removed. Cells were rinsed with cold PBS three times and then centrifuged, followed by removal of supernatants. Annexin-V-FITC, PI, and HEPES buffer solution were prepared into Annexin-V-FITC/PI solution at 1:2:50 based on the instructions of Annexin-V-FITC Apoptosis Detection kit (K201-100, Biovision, Mountain View, CA, United States). Every 100 μL staining solution was used to resuspend 1 × 10^6^ cells and mixed thoroughly, incubated for 15 min. Then, 1 ml HEPES buffer (PB180325, Procell, Wuhan, China) was added and mixed thoroughly. The fluorescence of FITC and PI was detected by 252 nm and 620 nm band-pass filters at the wavelength of 488 nm and the apoptosis was detected (Diaz et al., 2015).

### Nuclear/Cytosol Fractionation Assay

Cells were resuspended in Hypotonic buffer A [10 mM HEPES (pH 7.5), 0.5 mM DTT, 10 mM KCl, 1.5 mM MgCl2] which contained protease and RNase inhibitor (N8080119, Thermo Fisher, Waltham, MA, United States). After 10-min incubation on ice, cells were centrifuged at 1,000× *g* at 4°C for 10 min. The supernatants were centrifuged at 15,000× *g* for 15 min to obtain cytoplasm. The precipitation was rinsed two times with hypotonic buffer, resuspended in Hypotonic buffer B [10 mM HEPES (pH 7.5), 0.5 mM DTT, 10 mM KCl, 1.5mM MgCl2, 0.5% Non-idet P-40] and incubated at 4°C for 30 min with gentle rotation, followed by centrifugation at 6,000× *g* at 4°C for 10 min. After being rinsed with hypotonic buffer, the precipitation was resuspended in RIPA buffer [50 mM Tris HC1 (pH 7.5), 1500 mM KC1, 1% Non-idet P-40, 0.5% sodium deoxycholate, 0.1% SDS, 1 mM EDTA (pH 8)] containing protease and Rnase inhibitor, incubated at 4°C for 30 min with gentle rotation, and centrifuged at 15,000× *g* for 20 min. The supernatant was the nuclear part. Kcnq1ot1 expression was tested using Reverse Transcription-Quantitative Polymerase Chain Reaction (RT-qPCR) with GAPDH as cytoplasmic marker and U6 as a nuclear marker.

### Dual-Luciferase Reporter Assay

The target site sequence of NLRP3 mRNA 3′-UTR wild type (WT) and the mutant sequence (MUT) were synthesized. The pmiR-RB-REPORTTM plasmid (RiboBio, Guangzhou, Guangdong, China) was digested using restriction endonuclease, and the synthesized target gene sequence WT and MUT were, respectively, inserted into pmiR-RB-REPORTTM vectors. The correctly sequenced luciferase reporter plasmid WT and MUT were transfected with mimic-NC or miR-204-5p mimic into 293T cells. After 48 h of transfection, cells were harvested, lysed, and centrifuged for 3–5 min. The relative luminescence unit (RLU1) of firefly luciferase in the supernatants was detected using Firefly Luciferase Assay kit (RG005, Beyotime, Shanghai, China), and relative fluorescent value was obtained with the relative luminescence unit (RLU2) of Ranilla luciferase as an internal control. The experiment was repeated three times. The binding relation between Kcnq1ot1 and miR-204-5p was verified in the same way. The wild-type sequence or mutant sequence of Kcnq1ot1 was, respectively, inserted into pGL3-reporter vectors and transfected with miR-204-5p mimic or mimic-NC into the HEK293T cells, followed by detecting luciferase activity.

### RNA Immunoprecipitation Assay

The protein binding of Kcnq1ot1 and Ago2 was detected with a RNA Immunoprecipitation (RIP) kit (Millipore, Billerica, MA, United States). HK-2 cells were rinsed with pre-cooled PBS and supernatants were removed. Then, cells were lysed for 5 min with the same amount of RIPA lysis buffer and centrifuged for 10 min at 14,000 rpm at 4°C. The supernatants were coprecipitated with antibodies. A total of 50 μl magnetic beads used for each coprecipitation system was washed, resuspended with 100 μl RIP Wash Buffer, and then added with 5 μl of antibody for binding based on grouping design. The magnetic bead antibody complex was washed, followed by resuspension with 900 μl of RIP Wash Buffer and incubation with 100 μl of cell extracts overnight at 4°C. The sample was put on a magnetic stand to collect the magnetic bead antibody complex. RNA was extracted from samples after detachment using protease K for following RT-qPCR. Antibodies used in RIP assay were as follows: rabbit anti-Ago2 (ab186733, 1:50, Abcam) mixed for 30 min with rabbit anti-IgG (ab109489, 1:100, Abcam) as the negative control. The experiment was repeated three times.

### RNA-Pull Down

WT-bio-miR-204-5p or MUT-miR-204-5p (GeneCreate, Wuhan, China) labeled with 50 nM biotin was delivered into cells. Cells were harvested and rinsed with PBS after 48-h transfection and then mixed for 10 min with a specific lysis buffer (Ambion, Austin, TX, United States). The lysates were supplemented with M-280 streptavidin magnetic beads (S3762, Sigma) pre-coated with Rnase-free BSA and yeast tRNA (TRNABAK-RO, Sigma) at 4°C overnight, subsequently washed with lysis buffer two times, and washed three times with low salt buffer and once with high salt buffer. The binding RNA was purified by TRIzol and RT-qPCR was employed to detect the enrichment of Kcnq1ot1. The experiment was repeated three times.

### Statistical Analysis

All data were analyzed using SPSS 21 software (IBM Corp., Armonk, NY, United States). Data were described as *M* ± *SD*. Pairwise comparisons were analyzed using an independent sample *t*-test, while differences among multi-groups were analyzed using the one-way ANOVA, followed by Turkey’s test. Pairwise comparisons at different time points were analyzed using repeated-measures ANOVA, followed by Bonferroni’s test. *P* < 0.05 was regarded as statistically significant.

## Results

### Long Non-Coding RNA Kcnq1ot1 Was Highly Expressed in Mice With Acute Kidney Injury

**FIGURE 1:**
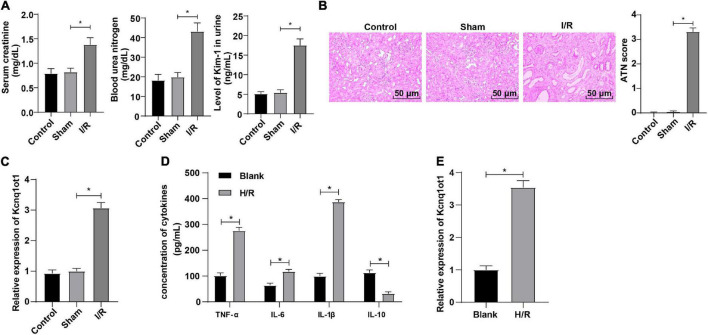
LncRNA Kcnq1ot1 was highly expressed in mice with AKI. **(A)** Levels of SCr, BUN, and KIM-1 were detected by ELISA; **(B)** pathological changes of renal tissues were detected by HE staining and quantitative analysis of tissue damage was performed; **(C)** Kcnq1ot1 expression in renal tissues was detected by RT-qPCR; **(D)** levels of TNF-α, IL-6, IL-1β, and IL-10 in the supernatants were detected by ELISA; **(E)** Kcnq1ot1 expression in H/R model of HK-2 cells was detected by RT-qPCR. All data in this figure were measurement data and presented as *M* ± *SD*. Comparison between two groups was analyzed using an independent sample *t*-test. *P < 0.05. The experiment was repeated three times.

To further explore the effect of Kcnq1ot1 on AKI, the AKI mouse model was established. Compared with the sham group, Enzyme-Linked Immunosorbent Assay (ELISA) showed that levels of SCr, BUN, and KIM-1 were increased in the I/R group (Figure 1A). HE staining showed that the kidney tubule was severely injured in the I/R group (Figure 1B). Reverse transcription-quantitative polymerase chain reaction showed that Kcnq1ot1 expression was increased in the I/R group (Figure 1C). No statistical differences were observed between the control group and the sham group. The H/R model of HK-2 cells was established in this study. ELISA showed that compared with the blank group, levels of TNF-α, IL-6, and IL-1β were increased while IL-10 level was decreased in the H/R group (Figure 1D). Reverse transcription-quantitative polymerase chain reaction showed that Kcnq1ot1 expression was increased in the H/R group compared with the blank group (Figure 1E). These results suggested that Kcnq1ot1 was highly expressed in the AKI model *in vivo* and *in vitro*.

### Kcnq1ot1 Knockdown Inhibited Inflammation in the H/R Model of HK-2 Cells *in vitro*

**FIGURE 2:**
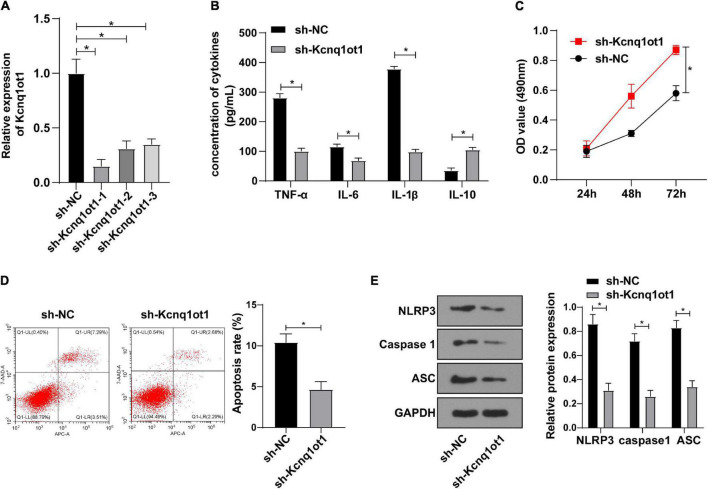
Kcnq1ot1 knockdown inhibited inflammation in the H/R model of HK-2 cells *in vitro*. **(A)** Transfection efficiency of Kcnq1ot1 in H/R model of HK-2 cells was detected by RT-qPCR; **(B)** levels of TNF-α, IL-6, IL-1β, and IL-10 in the supernatants were detected by ELISA; **(C)** cell viability was detected by CCK-8 assay; **(D)** cell apoptosis was detected by flow cytometry; **(E)** levels of NLRP3 inflammasome related factors were detected by Western blot. All data in this figure were measurement data and presented as *M* ± *SD*. Comparison between two groups was analyzed using an independent sample *t*-test, while comparison among groups at different time points was analyzed using the repeated measures ANOVA, followed by Bonferroni’s *post hoc* test. *P < 0.05. The experiment was repeated three times.

To identify the effect of Kcnq1ot1 on biological functions of HK-2 cells, Kcnq1ot1 in the H/R model of HK-2 cells was silenced and the transfection efficiency was verified by RT-qPCR (Figure 2A). ELISA showed that compared with the sh-NC group, levels of TNF-α, IL-6 and IL-1β were decreased while IL-10 level was increased in the sh-Kcnq1ot1 group (Figure 2B). CCK-8 assay showed that the ability of cell proliferation was significantly increased in the sh-Kcnq1ot1 group (Figure 2C). Flow cytometry showed that the apoptotic rate of cells in the sh-Kcnq1ot1 group was reduced compared with that of the sh-NC group (Figure 2D). NLRP3 knockdown improved the activity of renal tubular cells by alleviating myoglobin-induced mitochondrial damage and lipid peroxidation (Song et al., 2020). Western blot showed that compared with the sh-NC group, levels of NLRP3, caspase1 and ASC were decreased in the sh-Kcnq1ot1 group (Figure 2E). The above results indicated that inflammatory response was inhibited after silencing Kcnq1ot1 *in vitro* model.

### Kcnq1ot1 Could Competitively Bind to miR-204-5p and Inhibit miR-204-5p Expression

**FIGURE 3:**
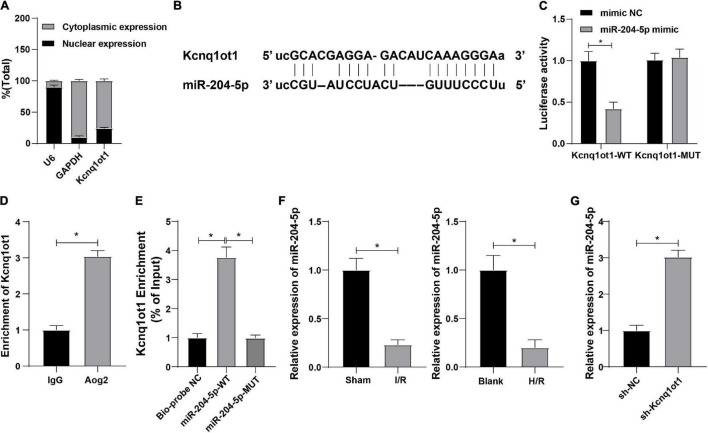
Kcnq1ot1 could competitively bind to miR-204-5p and inhibit miR-204-5p expression. **(A)** Subcellular localization of Kcnq1ot1 was observed by nuclear/cytosol fractionation assay with GAPDH as cytoplasm marker and U6 as a nuclear marker; **(B)** binding sites of Kcnq1ot1 and miR-204-5p were predicted through bioinformatics website Starbase; **(C)** binding relation between Kcnq1ot1 and miR-204-5p was verified by dual-luciferase reporter assay; **(D)** binding condition of Kcnq1ot1 and miR-204-5p was detected by RIP assay; **(E)** enrichment of Kcnq1ot1 by miR-204-5p was detected by RNA-pull down; **(F)** miR-204-5p expression in AKI mouse model and H/R model of HK-2 cells was detected by RT-qPCR; **(G)** miR-204-5p expression in HK-2 cells transfected with sh-Kcnq1ot1 was detected by RT-qPCR. Data were presented as *M* ± *SD*. Comparison between two groups was analyzed using an independent sample *t*-test, while comparison among multiple groups was analyzed using the one-way ANOVA, followed by Tukey’s *post hoc* test. *P < 0.05. The experiment was repeated three times.

To further study the functional mechanism of Kcnq1ot1 in AKI, nuclear/cytosol fractionation assay was adopted to identify the subcellular localization of Kcnq1ot1 and the result showed that Kcnq1ot1 was mainly located at the cytoplasm of HK-2 cell (Figure 3A), indicating Kcnq1ot1 may competitively bind to a miRNA in HK-2 cells to form a ceRNA regulatory network. The binding sites of Kcnq1ot1 and miR-204-5p were predicted through bioinformatics website Starbase^1^ (Figure 3B). Dual-luciferase reporter assay showed that the luciferase activity in the binding region of Kcnq1ot1-WT and miR-204-5p was suppressed while no evident difference of Kcnq1ot1-MUT luciferase activity was observed in the miR-204-5p mimic group (Figure 3C). RNA immunoprecipitation assay showed that anti-Ago2 antibody precipitated Kcnq1ot1 (Figure 3D). RNA-pull down assay showed that Kcnq1ot1 binding to miR-204-5p-WT was upregulated while Kcnq1ot1 binding to miR-204-5p-MUT showed no significant difference, suggesting a direct binding between miR-204-5p and Kcnq1ot1 (Figure 3E). Reverse transcription-quantitative polymerase chain reaction demonstrated that miR-204-5p was weak in the AKI mouse model and H/R cell model (Figure 3F, *P* < 0.05), and miR-204-5p was upregulated in the sh-Kcnq1ot1 group (Figure 3G, all *P* < 0.05). These results elucidated that Kcnq1ot1 inhibited miR-204-5p expression by competitively binding to miR-204-5p.

### miR-204-5p Overexpression Averted the Promotion of Kcnq1ot1 on Inflammation in the H/R Model of HK-2 Cells

**FIGURE 4:**
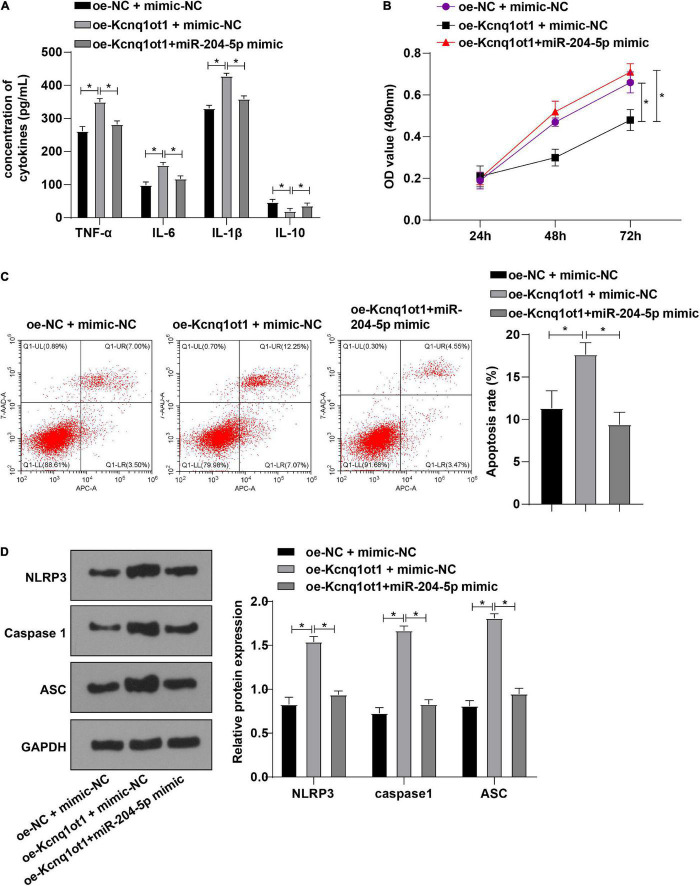
miR-204-5p overexpression averted the promotion of Kcnq1ot1 on inflammation in the H/R model of HK-2 cells *in vitro*. **(A)** Levels of TNF-α, IL-6, IL-1β, and IL-10 in the supernatants were detected by ELISA; **(B)** cell viability was detected by CCK-8 assay; **(C)** cell apoptosis was detected by flow cytometry; **(D)** levels of NLRP3 inflammasome related factors were detected by Western blot. All data in this figure were measurement data and presented as *M* ± *SD*. Comparison among multiple groups was analyzed using the one-way ANOVA and Turkey’s *post hoc* test while comparison among groups at different time points was analyzed using the repeated measures ANOVA, followed by Bonferroni’s *post hoc* test. *P < 0.05. The experiment was repeated three times.

To further unravel whether Kcnq1ot1 affects biological functions of HK-2 cells through miR-204-5p, HK-2 cells in the H/R model were transfected and assigned into the following groups: oe-NC + mimic-NC group, oe-Kcnq1ot1 + mimic-NC group, and oe-Kcnq1ot1 + miR-204-5p mimic group. ELISA showed that levels of TNF-α, IL-6, and IL-1β were increased while IL-10 level was decreased in oe-Kcnq1ot1 + mimic-NC group relative to oe-NC + mimic-NC group; levels of TNF-α, IL-6, and IL-1β were decreased while IL-10 level was increased in oe-Kcnq1ot1 + miR-204-5p mimic group relative to oe-Kcnq1ot1 + mimic-NC group (Figure 4A). CCK-8 assay showed that cell proliferation was repressed in oe-Kcnq1ot1 + mimic NC group relative to oe-NC + mimic-NC group; cell proliferation was promoted in oe-Kcnq1ot1 + miR-204-5p mimic group relative to oe-Kcnq1ot1 + mimic-NC group (Figure 4B). Flow cytometry showed that cell apoptosis was promoted in the oe-Kcnq1ot1 + mimic-NC group relative to oe-NC + mimic-NC group; cell apoptosis was inhibited in the oe-Kcnq1ot1 + miR-204-5p mimic group relative to oe-Kcnq1ot1 + mimic-NC group (Figure 4C). Western blot showed that expressions of NLRP3, caspase1, and ASC were upregulated in the oe-Kcnq1ot1 + mimic-NC group relative to oe-NC + mimic-NC group while expressions of NLRP3, caspase1, and ASC were decreased in oe-Kcnq1ot1 + miR-204-5p mimic group relative to oe-Kcnq1ot1 + mimic-NC group (Figure 4D). These results elicited that miR-204-5p overexpression annulled the promotion of Kcnq1ot1 on inflammation in the H/R model of HK-2 cells *in vitro*.

### NLRP3 Overexpression Averted Inhibitive Effect of miR-204-5p on Inflammation in HK-2 Cells

**FIGURE 5:**
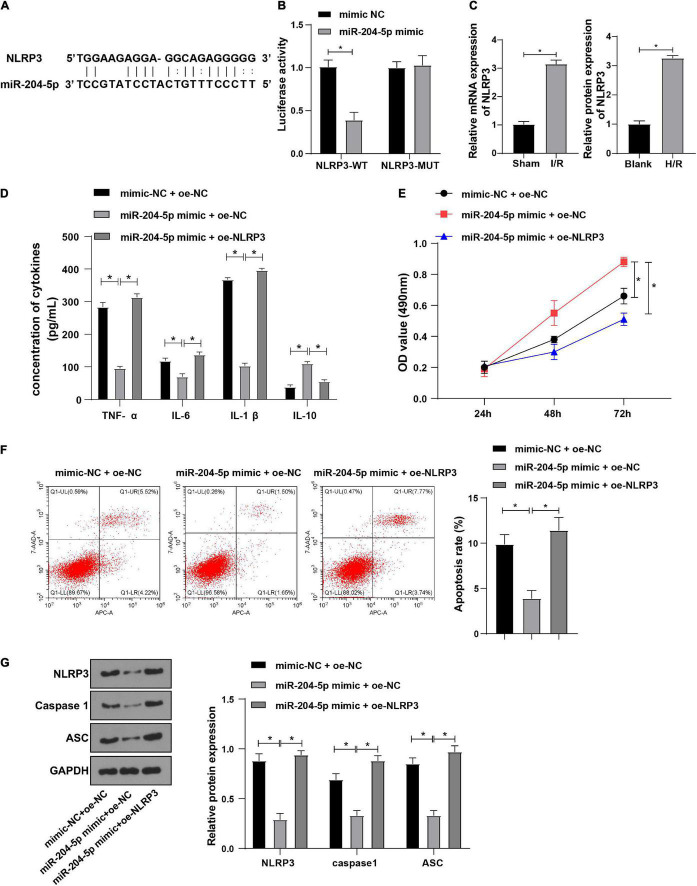
NLRP3 overexpression averted inhibitive effect of miR-204-5p on inflammation in H/R-induced HK-2 cells. **(A)** Target binding sites of miR-204-5p and NLRP3 were predicted through the RNA22 website; **(B)** target relation between miR-204-5p and NLRP3 was verified by dual-luciferase reporter assay; **(C)** NLRP3 expression in AKI mouse and H/R model of HK-2 cells was detected by RT-qPCR; **(D)** levels of TNF-α, IL-6, IL-1β, and IL-10 in the supernatants were detected by ELISA; **(E)** cell viability was detected by CCK-8 assay; **(F)** cell apoptosis was detected by flow cytometry; **(G)** levels of NLRP3 inflammasome related factors were detected by Western blot. All data in this figure were measurement data and presented as *M* ± *SD*. Comparison among multiple groups was analyzed using the one-way ANOVA and Turkey’s *post hoc* test while comparison among groups at different time points was analyzed using the repeated measures ANOVA, followed by Bonferroni’s *post hoc* test. *P < 0.05. The experiment was repeated three times.

The downstream target genes of miR-204-5p were investigated in the following experiment. NLRP3 was reported to alleviate sepsis-induced AKI (Zhang X. et al., 2021). The binding sites of miR-204-5p and NLRP3 were found through the RNA22 website (Figure 5A). Meanwhile, dual-luciferase reporter assay showed that miR-204-5p could specifically bind to NLRP3 (Figure 5B, all *P* < 0.05). Therefore, we hypothesized that Kcnq1ot1 could activate the NLRP3 inflammasome by sponging miR-204-5p as a ceRNA. Meanwhile, RT-qPCR showed that NLRP3 was highly expressed in the AKI mouse model and H/R model of HK-2 cells (Figure 5C, *P* < 0.05).

To further explore whether miR-204-5p affects the biological functions of HK-2 cells through NLRP3, HK-2 cells in the H/R model were transfected and assigned into the following groups: mimic-NC + oe-NC group, miR-204-5p mimic + oe-NC group and miR-204-5p mimic + oe-NLRP3 group. ELISA showed that levels of TNF-α, IL-6, and IL-1β were decreased while IL-10 level was increased in miR-204-5p mimic + oe-NC group relative to mimic-NC + oe-NC group; levels of TNF-α, IL-6, and IL-1β were increased while IL-10 level was decreased in miR-204-5p mimic + oe-NLRP3 group relative to miR-204-5p mimic + oe-NC group (Figure 5D). CCK-8 assay indicated that the ability of cell proliferation was increased in miR-204-5p mimic + oe-NC group relative to mimic-NC + oe-NC group while the ability of cell proliferation was decreased in miR-204-5p mimic + oe-NLRP3 group relative to miR-204-5p mimic + oe-NC group (Figure 5E). Flow cytometry showed that the apoptotic ratio was reduced in the miR-204-5p mimic + oe-NC group relative to the mimic-NC + oe-NC group while the apoptotic ratio was increased in the miR-204-5p mimic + oe-NLRP3 group relative to miR-204-5p mimic + oe-NC group (Figure 5F). Western blot showed that expressions of NLRP3, caspase1, and ASC were decreased in miR-204-5p mimic + oe-NC group compared with mimic-NC + oe-NC group while expressions of NLRP3, caspase1, and ASC were increased in miR-204-5p mimic + oe-NLRP3 group compared with miR-204-5p mimic + oe-NC group (Figure 5G). These results indicated that NLRP3 overexpression abolished the inhibitive effect of miR-204-5p on inflammation in HK-2 cells.

### *In vivo* Knockdown of Kcnq1ot1 Inhibited the Progression of Acute Kidney Injury by Regulating the miR-204-5p/NLRP3 Axis

**FIGURE 6:**
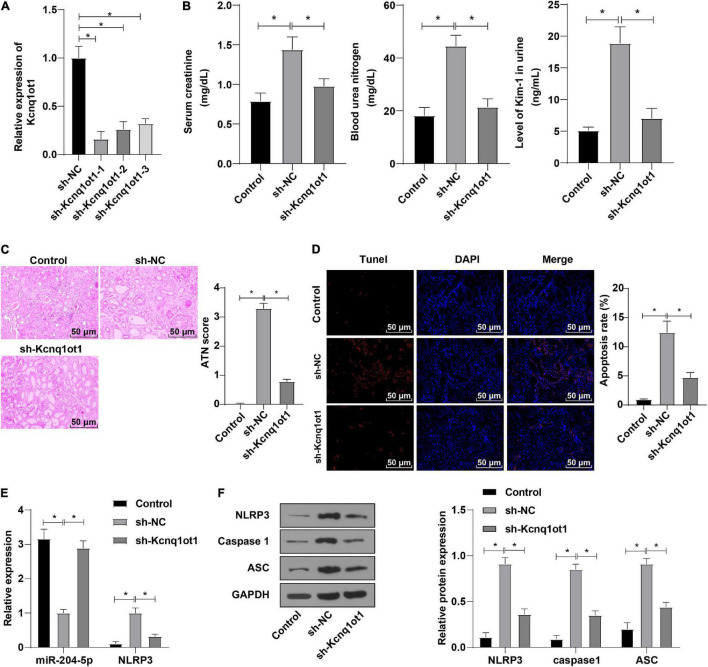
*In vivo* knockdown of Kcnq1ot1 inhibited the progression of AKI by regulating the miR-204-5p/NLRP3 axis. **(A)** Transfection efficiency after interfering Kcnq1ot1 was detected by RT-qPCR; **(B)** levels of SCr, BUN, and KIM-1 in mouse serum were detected by ELISA; **(C)** pathological changes of renal tissues were detected by HE staining and tissue damage degree was statistically analyzed; **(D)** cell apoptosis in renal tissues was detected by TUNEL staining; **(E)** expressions of miR-204-5p and NLRP3 in renal tissues were detected by RT-qPCR; **(F)** levels of NLRP3, caspase1, and ASC in renal tissues were detected by Western blot. All data in this figure were measurement data and presented as *M* ± *SD*. Comparison between two groups was analyzed using an independent sample *t*-test. *P < 0.05. The experiment was repeated three times.

To further validate whether Kcnq1ot1 affects AKI development by mediating the miR-204-5p/NLRP3 axis, mice were injected with the Kcnq1ot1 knockdown vector one week before establishing the AKI model and transfection efficiency was detected by RT-qPCR (Figure 6A). ELISA showed that compared with the sh-NC group, levels of SCr, BUN, and KIM-1 were significantly decreased in the sh-Kcnq1ot1 group (Figure 6B). Hematoxylin and eosin staining showed that renal tubular injury was mitigated in the sh-Kcnq1ot1 group (Figure 6C). Transferase dUTP nick end labeling staining showed that cell apoptosis was reduced after silencing Kcnq1ot1 expression (Figure 6D). Reverse transcription-quantitative polymerase chain reaction showed that miR-204-5p expression was upregulated while NLRP3 expression was downregulated in the sh-Kcnq1ot1 group (Figure 6E). Western blot showed that levels of NLRP3, caspase1, and ASC were greatly decreased in the sh-Kcnq1ot1 group (Figure 6F). The above results suggested that *in vivo* knockdown of Kcnq1ot1 inhibited AKI by modulating the miR-204-5p/NLRP3 axis.

The levels of SCr and BUM in the serum of mice were determined by ELISA at 1 and 2 weeks after establishing the AKI model. The results showed that the levels of SCr and BUN were significantly reduced after Kcnq1ot1 knockdown and no tendency to increase was observed over time, indicating the absence of extensive fibrosis (Supplementary Figure 1).

## Discussion

**FIGURE 7:**
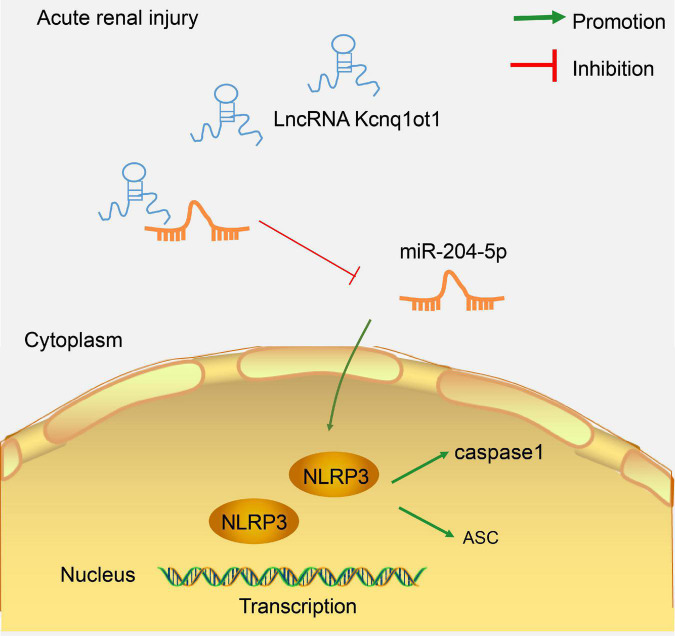
LncRNA Kcnq1ot1 mediated NLRP3 inflammasome activation by sponging miR-204-5p as ceRNA. The knockdown of lncRNA Kcnq1ot1 could inhibit NLRP3 inflammasome activation and thus inhibit the inflammatory response in AKI by blocking the sponging of miR-204-5p as ceRNA to increase miR-204-5p expression.

AKI is a frequent complication among hospitalized patients and exerts a detrimental effect on the outcomes (Peerapornratana et al., 2019). Long non-coding RNAs regulate a wide variety of biological activities in many organs including kidneys (Ren et al., 2019). In this study, we identified the mechanism of lncRNA Kcnq1ot1 in AKI by mediating NLRP3 inflammasome activation by sponging miR-204-5p (Figure 7).

A previous study reported that silencing Kcnq1ot1 attenuated inflammation in acute respiratory distress syndrome (Jiang et al., 2020), yet the mechanism of Kcnq1ot1 in AKI remains unclear. Kcnq1ot1 was notably elevated in diabetic nephropathy and Kcnq1ot1 knockdown reduced inflammation and pyroptosis in high glucose-induced HK-2 cells (Zhu et al., 2020). In this study, the AKI mouse model was established to identify the effect of Kcnq1ot1 on AKI. SCr and BUN are important for the prediction of chronic kidney disease of any stage and KIM-1 is a renal tubular injury marker (Hussein et al., 2020; Qi et al., 2020). Our result showed that levels of SCr, BUN, and KIM-1 were increased, and renal tubular injury was exacerbated in I/R mice, and Kcnq1ot1 was elevated in I/R mice. Pro-inflammatory cytokines play key roles in kidney injury while anti-inflammatory cytokine IL-10 is implicated in AKI treatment (Zhang et al., 2019; Deng et al., 2020). The H/R model of HK-2 cells was established in this study and the result showed that levels of TNF-α, IL-6, and IL-1β were enhanced while IL-10 level was weak in the H/R cell model and Kcnq1ot1 expression was strong in the H/R cell model. Furthermore, Kcnq1ot1 was silenced to further explore its effect on the biological functions of cells. Our results showed that TNF-α, IL-6, and IL-1β levels were decreased while IL-10 level was increased after silencing Kcnq1ot1, the ability of cell proliferation was promoted, while apoptosis was decreased after silencing Kcnq1ot1. Consistently, silencing Kcnq1ot1 inhibits inflammation in cardiac fibroblasts (Yang et al., 2018a). Kcnq1ot1 knockdown restrains cell proliferation and induces apoptosis in diabetic nephropathy (Jie et al., 2020). NLRP3, caspase1, and ASC are pyroptosis-related genes and Kcnq1ot1 knockdown reverses the high level of NLRP3 in diabetic nephropathy, inhibits caspase1 in cardiomyocytes (Yang et al., 2018b; Zhu et al., 2020). In our study, expressions of NLRP3, caspase1, and ASC were decreased in the H/R model of HK-2 cells after silencing Kcnq1ot1. Silencing Kcnq1ot1 was reported to mitigate inflammatory response in myocardial infarction (Wang et al., 2019). Therefore, we concluded that knockdown of Kcnq1ot1 suppresses inflammatory response *in vitro*.

To further investigate the functional mechanism of Kcnq1ot1 in AKI, the subcellular localization of Kcnq1ot1 was identified and the result showed that Kcnq1ot1 was mainly located at the cytoplasm of HK-2 cell, which suggested that Kcnq1ot1 may competitively bind to a miRNA in HK-2 cells to form a ceRNA regulatory network. miR-204-5p is implicated in renal I/R injury (Zhu et al., 2019). miR-204-5p is inversely correlated with Kcnq1ot1 (Kang et al., 2019). In our study, Kcnq1ot1 binding to miR-204-5p-WT was upregulated while Kcnq1ot1 binding to miR-204-5p-MUT was downregulated. Bioinformatics website, dual-luciferase reporter assay, RIP assay and RNA-pull down suggested a direct binding between miR-204-5p and Kcnq1ot1. miR-204-5p was downregulated in patients with hypertension, hypertensive nephrosclerosis, or diabetic nephropathy (Cheng et al., 2020). Additionally, the result showed that miR-204-5p was weakly expressed in the AKI rat model and cell model, and miR-204-5p was upregulated after silencing Kcnq1ot1. Collectively, Kcnq1ot1 inhibited miR-204-5p expression by competitively binding to miR-204-5p.

The results further showed that miR-204-5p has been documented to suppress IL-6-mediated inflammatory response in HK-2 renal tubular epithelial cells (Li et al., 2019). Hence, whether Kcnq1ot1 affects biological functions of HK-2 cells via miR-204-5p was explored next. miR-204-5p or/and Kcnq1ot1 was overexpressed based on the H/R model of HK-2 cells. In our study, levels of TNF-α, IL-6, and IL-1β were decreased while IL-10 level was increased in HK-2 cells transfected with oe-Kcnq1ot1 + miR-204-5p mimic relative to oe-Kcnq1ot1 + mimic NC. miR-204 overexpression reduces expressions of TNF-α, IL-1β, and IL-6 in myocardial I/R injury (Xue and Luo, 2019). Besides, cell proliferation was promoted, cell apoptosis was inhibited, and expressions of NLRP3, caspase1, and ASC were decreased in HK-2 cells transfected with oe-Kcnq1ot1 + miR-204-5p mimic compared with HK-2 cells transfected with oe-Kcnq1ot1 + mimic NC. miR-204-5p promotes cell proliferation in human adipose-derived mesenchymal stem cells (Li D. et al., 2020). miR-204-5p could inhibit the inflammatory response in lipopolysaccharide-treated rat mesangial cells (Chen et al., 2018). Taken together, miR-204-5p overexpression averted the promoting effect of Kcnq1ot1 on inflammatory response in HK-2 cell injury model *in vitro*.

The downstream targets of miR-204-5p were explored subsequently. NLRP3 was one of the targets of miR-204-5p. NLRP3 inflammasome is essential in the pathogenesis of contrast-induced AKI (Lin et al., 2019). The binding sites of miR-204-5p and NLRP3 were predicted and specific binding of miR-204-5p to NLRP3 was verified. It was hypothesized that Kcnq1ot1 activated NLRP3 inflammasome by sponging miR-204-5p as a ceRNA. The result elicited that NLRP3 was highly expressed in AKI models, consistent with a previous study that NLRP3 expression was elevated in renal I/R injury (Sun et al., 2020). However, whether NLRP3 participated in the regulation of miR-204-5p on HK-2 cells remained unknown. miR-204-5p or/and NLRP3 was overexpressed based on the H/R model of HK-2 cells. In our study, levels of TNF-α, IL-6, and IL-1β were elevated while IL-10 level was reduced in HK-2 cells delivered with miR-204-5p mimic + oe-NLRP3 relative to miR-204-5p mimic + oe-NC. Decreased inflammation could be traced to decreased NLRP3 expression in bone marrow chimera studies (Mao et al., 2018). Besides, the ability of cell proliferation was decreased, while apoptosis was increased in HK-2 cells transfected with miR-204-5p mimic + oe-NLRP3 relative to miR-204-5p mimic + oe-NC. Overexpression of NLRP3 inhibits cell proliferation and stimulates apoptosis (Salaro et al., 2016; Tan et al., 2020). The NLRP3 inflammasome consists of NLRP3, ASC, and caspase1 (Salaro et al., 2016). Our study result showed that expressions of NLRP3, caspase1, and ASC were elevated in HK-2 cells transfected with miR-204-5p mimic + oe-NLRP3 compared with miR-204-5p mimic + oe-NC. NLRP3 inflammasome promotes renal inflammation in chronic kidney diseases (Chen et al., 2017). The inhibition of NLRP3 alleviates the promoting effect of miR-200a-3p on inflammation in sepsis (Yu et al., 2019). Therefore, NLRP3 overexpression averted the inhibitive effect of miR-204-5p on inflammatory response in HK-2 cells.

To further validate whether Kcnq1ot1 affects AKI rats through the miR-204-5p/NLRP3 axis, AKI mice were injected with Kcnq1ot1 knockdown vectors. The result showed that levels of SCr, BUN, and KIM-1 were significantly decreased, and renal tubular injury was mitigated after Kcnq1ot1 knockdown. miR-204-5p expression was upregulated while NLRP3 expression was downregulated and levels of NLRP3, caspase1, and ASC were greatly decreased after Kcnq1ot1 knockdown. Kcnq1ot1 induces sC5b-9-induced podocyte pyroptosis by reducing miR-486a-3p and enhancing NLRP3 (Zhang C. et al., 2021). Kcnq1ot1 knockdown and miR-506-3p overexpression reduce pyroptosis by downregulating NLRP3 expression in diabetic nephropathy (Zhu et al., 2020). Consistently, Kcnq1ot1 knockdown inhibited the inflammatory response in AKI by modulating the miR-204-5p/NLRP3 axis.

## Conclusion

This study supported that the knockdown of lncRNA Kcnq1ot1 inhibited inflammation in AKI by blocking the sponging to miR-204-5p and limiting the activation of the NLRP3 inflammasome, and thus provided potential molecular targets for AKI treatment. Yet, this study simply showed the molecular mechanism of Kcnq1ot1 in AKI at the cellular and animal levels. Further studies are needed to investigate other miRNAs and target genes involved to provide strong support for these findings.

## Data Availability Statement

The original contributions presented in the study are included in the article/Supplementary Material, further inquiries can be directed to the corresponding author.

## Ethics Statement

The studies involving human participants were reviewed and approved by The First People’s Hospital of Shangqiu. The patients/participants provided their written informed consent to participate in this study. The animal study was reviewed and approved by The First People’s Hospital of Shangqiu. Written informed consent was obtained from the individual(s) for the publication of any potentially identifiable images or data included in this article.

## Author Contributions

JW contributed to the study concepts, study design, and definition of intellectual content. PJ contributed to the literature research, data analysis, and statistical analysis. XW contributed to the manuscript preparation. YZ contributed to the manuscript editing and review. JW and YZ contributed to the experimental studies and data acquisition. All authors read and approved the final manuscript.

## Conflict of Interest

The authors declare that the research was conducted in the absence of any commercial or financial relationships that could be construed as a potential conflict of interest.

## Publisher’s Note

All claims expressed in this article are solely those of the authors and do not necessarily represent those of their affiliated organizations, or those of the publisher, the editors and the reviewers. Any product that may be evaluated in this article, or claim that may be made by its manufacturer, is not guaranteed or endorsed by the publisher.

## Footnotes

1
http://starbase.sysu.edu.cn/agoClipRNA.php?source=mRNA

## Supplementary Material

Supplementary Figure 1 The levels of SCr and BUN in the serum of mice were determined by ELISA at 1 and 2 weeks after establishing the AKI model. All data in this figure were measurement data and presented as *M* ± *SD*. Comparison between two groups was analyzed using an independent sample *t*-test. ^∗^*P* < 0.05. ns, *P* > 0.05. The experiment was repeated three times.
