# Supplementary figures and images for: Silencing Long Non-coding RNA Kcnq1ot1 Limits Acute Kidney Injury by Promoting miR-204-5p and Blocking the Activation of NLRP3 Inflammasome
Source: Front Physiol. 2021 Nov 11;12:721524. doi: 10.3389/fphys.2021.721524 (PMC8632456; doi:10.3389/fphys.2021.721524)

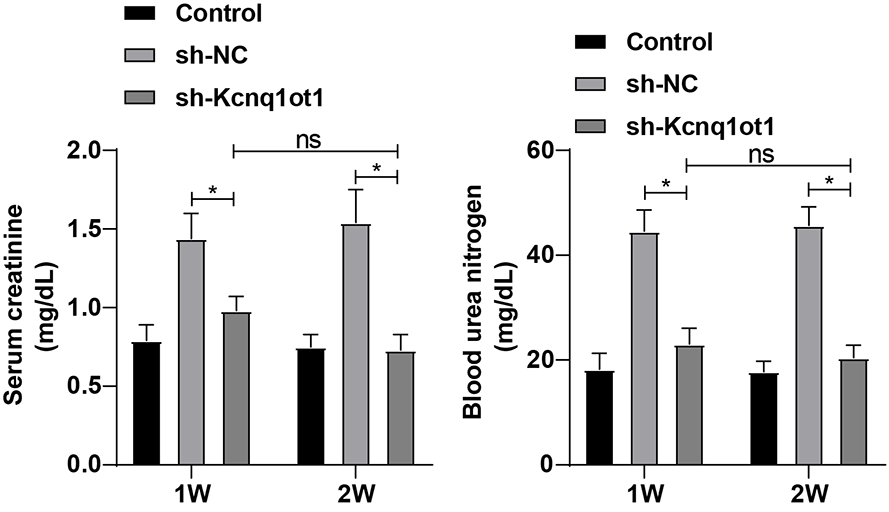

Supplement: Supplementary Figure 1 — The levels of SCr and BUN in the serum of mice were determined by ELISA at 1 and 2 weeks after establishing the AKI model. All data in this figure were measurement data and presented as M ± SD. Comparison between two groups was analyzed using an independent sample t-test. ∗P < 0.05. ns, P > 0.05. The experiment was repeated three times. [file Image_1.tiff]
